# Minor Change of Plasma Renin Activity during the Saline Infusion Test Provide an Auxiliary Diagnostic Value for Primary Aldosteronism

**DOI:** 10.1155/2021/5757305

**Published:** 2021-02-17

**Authors:** Munire Adilijiang, Qin Luo, Menghui Wang, Delian Zhang, Xiaoguang Yao, Guoliang Wang, Keming Zhou, Nanfang Li

**Affiliations:** Hypertension Center of People's Hospital of Xinjiang Uygur Autonomous Region, Xinjiang Hypertension Institute, National Health Committee Key Laboratory of Hypertension Clinical Research, Urumqi, China

## Abstract

**Objective:**

To clarify whether it has some hidden diagnostic values for PA, especially in the case of an inconclusive SIT result, we investigated the difference in changes of plasma renin activity (PRA) during SIT between patients with PA and non-PA.

**Methods:**

We measured and compared the SIT parameters of 159 PA patients, 368 non-PA patients, and 43 inconclusive patients who were included in this study.

**Results:**

The PA group showed a minor change of PRA during the SIT (ΔPRA, defined as (pre-SIT PRA–post-SIT PRA)) compared with the non-PA group (0.17 ng/ml/h vs. 1.07 ng/ml/h, *P* < 0.001). According to ROC analysis, ΔPRA showed a greater AUC than post-SIT PRA (0.897 vs. 0.855, *P* < 0.001). The cutoff value was 0.5 ng/ml/h, with 90.3% sensitivity and 78.6% specificity. When combined with ARR post-SIT, it showed 81.6% sensitivity and 97.0% specificity for PA diagnosis. Further analysis of 43 patients with an inconclusive SIT result who completed AVS found that ΔPRA was smaller in the confirmed PA group compared with the unconfirmed PA group (0.19 ng/ml/h vs. 0.29 ng/ml/h, *P* < 0.05); there was no significant difference in PAC post-SIT between two groups. ΔPRA ≤ 0.21 ng/ml/h provides 71.4% sensitivity, 80.0% specificity, and 87.0% PPV for their PA diagnosis.

**Conclusions:**

PA patients show minor PRA change during SIT; the change of PRA during SIT provides an auxiliary diagnostic value for PA, especially in patients with an inconclusive SIT result.

## 1. Introduction

Primary aldosteronism (PA), characterized by autonomous production of aldosterone and suppression of the renin, is a major cause of secondary hypertension accounting for up to 10%–15% of patients with hypertension [1–3]. As a potentially curable and specifically treatable form of secondary hypertension, PA represents a higher rate of ensuing cardiovascular events, renal damage, and metabolic syndrome than does essential hypertension (EH) [4–10]. Hence, early diagnosis and aggressive treatment are keys to managing PA and preventing its complications. For the detection of primary aldosteronism (PA), hypertensive patients are screened using the aldosterone-to-renin ratio (ARR). An elevated ratio requires confirmatory testing [10].

The saline infusion test (SIT) is the most commonly used confirmatory test recommended by the Endocrine Society guideline [10–13]. Suppression of plasma aldosterone concentration (PAC) after saline infusion is assumed to exclude PA. The Endocrine Society guideline suggests the use of two cutoff values: a post-SIT PAC >10 ng/dl confirms PA, whereas a post-SIT PAC <5 ng/dl excludes the diagnosis [10]. A post-infusion PAC from 5 to 10 ng/dl is referred to as inconclusive. In such cases, a diagnosis of PA is arbitrarily established or rejected based on contextual clinical and biochemical parameters.

The levels of plasma renin activity (PRA) and PAC decreased in response to rapid blood volume expansion and sodium loading in patients with essential hypertension during SIT [14]. The PAC of patients with PA could be insufficiently suppressed during SIT [15], whereas the suppression degree of their suppressed PRA during SIT was not focused by the previous study. Previous research reported that the suppressed plasma renin levels remained unchanged by saline infusion in patients with aldosterone-producing adenoma (APA) [16]. PA patients with suppressed plasma renin levels may have different renin changes during SIT compared with non-PA patients. And we postulate that changes in renin during SIT may have some hidden diagnostic values in the diagnosis of PA. Our study aimed at exploring the difference in changes of PRA during SIT between patients with PA and non-PA and to clarify whether it has some hidden diagnostic values in the diagnosis of PA, especially in patients with inconclusive SIT results.

## 2. Materials and Methods

### 2.1. Patients

As a retrospective cross-sectional study, the study was approved by the Ethics Committee of People's Hospital of Xinjiang Uygur Autonomous Region.

The subjects were selected from consecutive inpatients of the Hypertension Center of People's Hospital of Xinjiang Uygur Autonomous Region between January 2009 and December 2010 when the SIT was performed in those who had normal-high PAC (≥12 ng/dL) to identify renin-independent or renin-induced aldosteronism. We aimed at exploring the diagnostic value of PRA change during SIT for PA.

**Figure 1:**
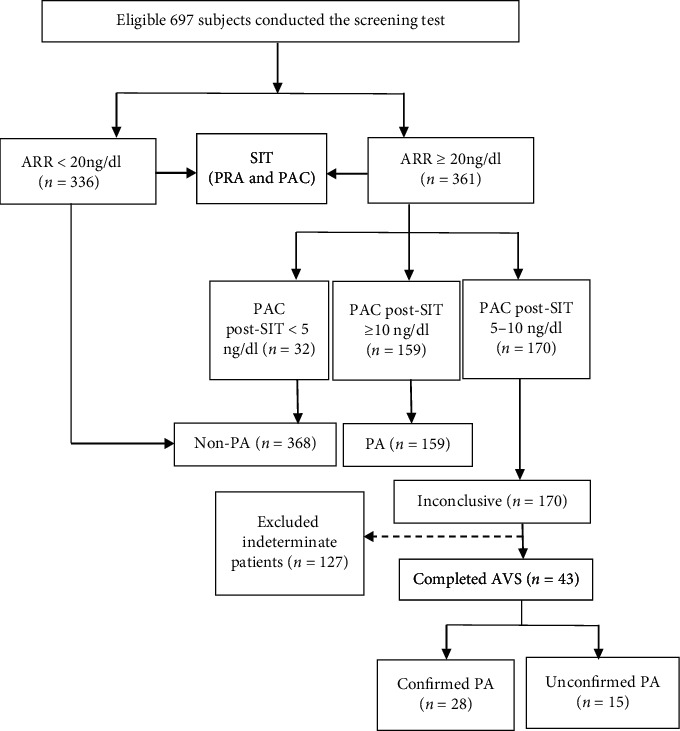
The procedure of subject recruitment. ARR, aldosterone-to-renin ratio; PA, primary aldosteronism; PAC, plasma aldosterone concentration; PRA, plasma renin activity; SIT, saline infusion test.

697 patients with seated PAC ≥ 12 ng/dl completed SIT and measured PRA together with PAC before and after SIT. 159 patients are diagnosed with PA, 368 patients are diagnosed with non-PA, and 170 patients are labelled as “Inconclusive.” Among the 170 patients with an inconclusive SIT result labelled as “Inconclusive,” 43 patients who were strongly willing to undergo adrenalectomy or who were highly suspected for PA by experienced hypertension experts based on their contextual clinical and biochemical parameters underwent AVS with successful cannulation of the adrenal veins (SI > 2). 28 patients of them were reclassified as “Confirmed PA,” including 11 unilateral PA and 17 bilateral PA, and 15 patients of them were reclassified as “Unconfirmed PA;” the remaining 127 patients considered as indeterminate patients were excluded from the study and received effective medical treatment and were followed up at our center. Finally, 159 subjects with PA, 368 subjects with non-PA, and 43 subjects with inconclusive SIT results (28 subjects with confirmed PA and 15 subjects with unconfirmed PA) enrolled in the present study (Figure 1).

### 2.2. Screening and Confirmatory Tests

All patients included completed the screening test and underwent SIT. Prior to testing, angiotensin-converting enzyme inhibitors, angiotensin receptor blockers, dihydropyridines calcium antagonist, and *β*-receptor blockers were always ceased for at least 4 weeks, and diuretics and mineralocorticoid antagonists were always ceased for at least 6 weeks or any antihypertensive medications were not taken at least 2 weeks. Wherever necessary, antihypertensive agents were replaced with slow-release verapamil or *α-*1 adrenergic antagonists (doxazosin or terazosin) or with a combination of the two to minimize the interference with the measurement of PAC, PRA, and ARR. And the abovementioned was maintained until SIT completed. Initial conditions of salt intake were estimated by 24-h urinary sodium measured prior to ARR as the basis of adjustment for salt intake. During the screening test, serum potassium had been monitored; patients who suffered from hypokalemia were corrected with oral potassium supplements, as close as possible to 3.9–4.0 mmol/l, and all subjects were recommended to maintain a full diet with liberal dietary salt intake (at least 6 g of NaCl per day). Blood samples were collected in the morning after patients have been ambulant for at least 2 h and seated for 15 min. In the process of SIT, patients remained in the seated position for 30 min before and during the infusion of 2 L of sodium chloride 0.9% over 4 hours. PRA and PAC were assessed before and after SIT.

The screening test was considered positive when ARR ≥ 20 (ng/dL)/(ng/mL/h). Our diagnosis was based on the saline infusion test (SIT) criteria in accordance with the Endocrine Society guideline [10]. Patients with the positive screening test and post-infusion PAC > 10 ng/dl were considered to “PA,” with the positive screening test and postinfusion PAC between 5 and 10 ng/dl labelled as “Inconclusive,” and those with the negative screening test or postinfusion PAC < 5 ng/dl were considered to be negative for PA. Patients labelled as “Inconclusive SIT” were diagnosed in PA or non-PA based on AVS results. AVS was performed without ACTH stimulation [10], and a diagnosis of aldosterone hypersecretion made when the aldosterone concentrations in the adrenal venous effluents were more than 250 ng/dl [17, 18]. Endocrine Society guidelines recommendations were referenced for criteria of lateralization and successful AVS [10]; successful AVS was defined as SI > 2, and lateralization was defined as LI ≥ 2. Patients with aldosterone hypersecretion on either side of the adrenal were classified as confirmed PA, while patients without aldosterone hypersecretion on both adrenals were classified as unconfirmed PA. 10 patients with lateralization (LI ≥ 2) in successful AVS among confirmed PA were patients diagnosed as unilateral PA, and 18 patients without lateralization (LI ≥ 2) in successful AVS among confirmed PA were patients diagnosed as bilateral PA.

### 2.3. Measurements

The office BP was determined by calculating the average from 3 measurements in the semirecumbent position after a 5-minute rest period [19]. The sodium and potassium levels were measured using standard methods. Hypokalemia was defined as serum potassium concentrations below 3.5 mmol/l. Serum and 24-h urinary sodium, potassium, and creatinine levels were measured on a C16000 automated biochemistry analyser (Abbott Laboratories, Abbott Park, IL, USA). Secondary causes of hypertension other than PA, such as Cushing syndrome, renal parenchymal disease, pheochromocytoma, renovascular hypertension, reninoma, hyperthyroidism, and so on, were excluded on the basis of laboratory analyses, plasma metanephrines and norepinephrine, renal artery duplex ultrasound or angiography, renal isotope scanning and overnight dexamethasone suppression testing, and so on, as clinically indicated. All patients underwent an abdominal computed tomographic scan or magnetic resonance imaging to assess adrenal morphology.

Plasma aldosterone was measured by radioimmunoassay using a commercially available kit (Beckman Coulter, Brea, CA, USA), and the intra and interassay coefficients of variation were 5.6 and 8.5%, respectively. PRA was measured by an iodine angiotensin I radioimmunoassay kit (Northern Biotechnology Institutes, Beijing, China), and the intra and interassay coefficients of variation were below 10% and 15%, respectively. The accuracy of detection of PRA could be affected by many factors such as posture, time of day, certain drugs including antihypertensives, age, assay reliability, and so on [20, 21]. All of above factors have been minimized by means of strict quality control in the full process of the ARR test and saline infusion test and multiple measurements of indicators. The assays of indicators such as PRA have been repeated at least three times. For calculation of ARR, the lowest value of PRA was set at 0.1 ng/mL/h.

### 2.4. Statistical Analysis

All statistical analyses were performed with SPSS statistical software, version 19.0 (Chicago, IL, USA). The data were expressed as mean ± S.D. or median (interquartile range). Data between groups were compared using Student's *t*-test, and multivariate analysis of variance for continuous variables and the LSD test were performed to estimate the differences between groups, and the *χ*^2^ test was performed for categorical variables. Receiver operating characteristic (ROC) curves were used to evaluate the accuracy and cutoff value of post-SIT PRA to diagnose PA. A *P* value of <0.05 was considered as statistically significant.

## 3. Results and Discussion

### 3.1. Baseline Characteristics

Clinical and biochemical characteristics of 570 subjects are summarized in Table 1. The proportion of patients with positive results in the screening test is 41%. The proportion of PA in this population was 32%. The average age was 43.8 ± 9 years, and the body mass index (BMI) was 27.1 ± 3.6 kg/m^2^. The systolic blood pressure (SBP) was 143 ± 19.5 mmHg, and diastolic blood pressure (DBP) was 97 ± 13.8 mmHg. The median PAC was 17.8 ng/dl (range: 14.5–23.7), the median PRA was 1.19 ng/ml/h (range: 0.42–2.49), and the median ARR was 15.0 (ng/dl)/(ng/ml/h) (range: 7.6–44.0). Serum potassium was 3.69 ± 0.38 mmol/L, and the incidence of hypokalemia was 27.0% (Supplementary Table 1).

Reported values are the number of patients with available data and then number (percentages) or median (first quartile and third quartile) or mean ± standard deviation. ARR, aldosterone-to-renin ratio; BMI, body mass index; PA, primary aldosteronism; PAC, plasma aldosterone concentration; PRA, plasma renin activity; serum *K*^+^, concentration of serum potassium; SIT, saline infusion test. ΔPAC = PAC pre-SIT−PAC post-SIT; ΔPRA = PRA pre-SIT−PRA post-SIT; PAC post-SIT/ΔPRA = PAC post-SIT/(PRA pre-SIT−PRA post-SIT).

### 3.2. Comparison of PRA Suppression in the SIT between the PA Group and Non-PA Group

There was no significant difference in gender proportion and body mass index between the PA group and non-PA group (*P* > 0.05). The age of the PA group is older than the non-PA group. Compared with the non-PA group, the PA group presented higher PAC and ARR, lower PRA, lower serum potassium level, higher proportion of hypokalemia, and higher proportion of adrenal mass or hyperplasia on CT (*P* < 0.05). Underwent SIT, the PA group still presented significantly higher post-SIT PAC (13.7 vs. 5.8 ng/dl, *P* < 0.05) and post-SIT ARR (68.6 vs. 12.9, *P* < 0.05) and significantly lower PRA post-SIT (0.20 vs.0.66 ng/ml/h, *P* < 0.05) compared with the non-PA group. Suppression of ΔPRA during SIT (ΔPRA defined as (PRA pre-SIT–PRA post-SIT)) in the PA group was significantly lower than that in the non-PA group. The ΔPRA of the two groups was 0.16 ng/ml/h and 1.07 ng/ml/h, respectively (*P* < 0.001). (Table 1).

**Figure 2:**
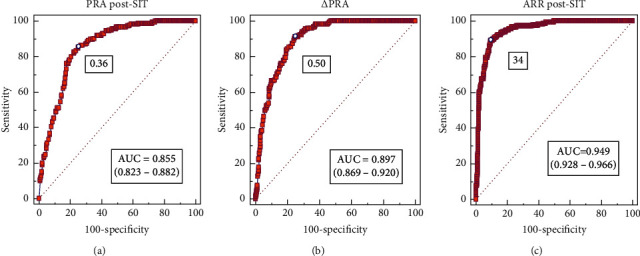
Receiver-operator characteristic (ROC) curves of PRA post-SIT, ΔPRA, and ARR post-SIT for primary aldosteronism (PA) diagnosis. PRA post-SIT (a), ROC curve of plasma renin activity after the saline infusion test; ΔPRA (b), ROC curve of reduction in PRA during the saline infusion test; and post-SIT ARR (c), ROC curve of aldosterone-renin ratio after the saline infusion test to diagnose PA. AUC indicates the area under the receiver-operator characteristics curve, and ΔPRA indicates reduction in PRA during SIT and diagnostic accuracy of ΔPRA, PRA post-SIT, and ARR post-SIT for PA.

**Figure 3:**
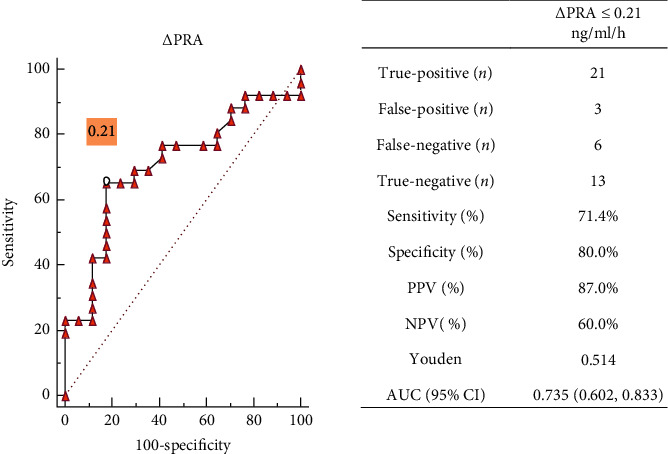
Receiver-operator characteristic (ROC) curves of ΔPRA for PA diagnosis among 43 patients with an inconclusive SIT result (28 with and 15 without PA) and relabelled by AVS. The optimal cutoff value was obtained according to the highest Youden index (YI): ΔPRA ≤ 0.21 ng/ml/h (sensitivity = 71.4%; specificity = 80.0%; PPV = 87.0%; NPV = 60.0%; YI = 0.514).

ROC analysis was undertaken to compare the diagnostic accuracy of ΔPRA, PRA post-SIT, and ARR post-SIT by comparing the areas under the ROC curves. For all of ΔPRA, PRA post-SIT, and ARR post-SIT, the area under the curve (AUC) was significantly (*P* < 0.001) greater than that under the reference line (AUC = 0.5) and greater than the AUC for PAC (0.97 ± 0.01, *Z* = 3.26, 1.94, 7.49, *P* < 0.05) (Supplementary Figure 1). The AUC for ΔPRA (0.90 ± 0.02) was greater than that for PRA post-SIT (0.85 ± 0.03; *Z* = 2.64, *P* < 0.05), and the AUC for ARR post-SIT (0.97 ± 0.01) was greater than that for ΔPRA (0.90 ± 0.02; *Z* = 4.48, *P* < 0.001) (Figure 2). The optimal cutoff value was obtained according to the highest Youden index (YI): ΔPRA≤0.5 ng/ml/h (sensitivity = 90.27%; specificity = 75.58%; YI = 0.659; PPV = 64%, NPV = 96.4%), post-SIT PRA<0.36 ng/ml/h (sensitivity 75.8%; specificity 69.1%; YI = 0.602, with high NPV of 93.1%), and post-SIT ARR>34 (sensitivity = 89.2%; specificity = 90.65%; PPV = 82.1%; NPV 94.6%; YI = 0.798) (Figure 3, Table 2). Of all the parameters, post-SIT other than PAC, PRA, ARR, and ΔPRA was most discriminatory between PA and non-PA groups. Therefore, these renin relevant parameters were combined in various permutations to calculate the diagnostic values for PA. The combination of ΔPRA ≤ 0.5 ng/ml/h and post-SIT ARR > 34 superior to all other parameters, with sensitivity of 81.6%, specificity of 97%, PPV of 92.6%, NPV of 91.6%, +*L* of 21, and ‒L of 0.15, which improve the specificity of ARR post-SIT from 90% to 97% and the PPV from 82.8% to 92.6%, maintain a similar NPV (Table 2).

### 3.3. Analysis in 43 Patients with Inconclusive SIT Results and Completed AVS

43 patients with an inconclusive test result in SIT (post-SIT PAC rang 5–10 ng/dl) was divided into the confirmed PA group (*n* = 28) and the unconfirmed PA group (*n* = 15) according to AVS. The confirmed PA group included 10 UPA and 18 IHA. No differences in age and body mass index was observed between the confirmed PA and unconfirmed PA groups (*P* < 0.05). SIT, PAC post-SIT, PRA post-SIT, and ARR post-SIT have no differences in two groups (*P* < 0.05). While, the PA group showed a smaller ΔPRA compared with the non-PA group (0.16 ng/ml/h vs. 0.29 ng/ml/h, respectively) (*P* < 0.05) (Table 3).

ROC analysis was undertaken to evaluate the diagnostic accuracy of ΔPRA for PA among 43 patients with inconclusive SIT results and completed AVS (28 patients with confirmed PA in AVS and 15 patients with unconfirmed PA in AVS); the area under the curve (AUC) was 0.735 (0.602, 0.833) and significantly (*P* < 0.001) greater than that under the reference line (AUC = 0.5). The optimal cutoff value was obtained according to the highest Youden index (YI): ΔPRA ≤ 0.21 ng/ml/h (sensitivity = 71.4%; specificity = 80.0%; PPV = 87.0%; NPV = 60.0%; YI = 0.514). Figure 3 shows the diagnostic efficacy for PA diagnosis in patients with inconclusive SIT results when ΔPRA ≤ 0.21 ng/ml/h was used as the judgment indicator. There were 21 cases of true-positive PA and 13 cases of true-negative non-PA; 79.1% (34/43) of patients with inconclusive SIT results were correctly diagnosed by ΔPRA (Figure 3).

## 4. Discussion

In the present study, we retrospectively analysed clinical data of 159 PA patients, 368 non-PA patients, and 43 inconclusive patients and found that PA patients show a minor PRA change during SIT; the change of PRA during SIT has been of the diagnostic value for PA, especially in patients with an inconclusive SIT result. Our results showed that patients who have a post-SIT ARR more than 34 and ΔPRA less than 0.5 ng/ml/h should be confirmed as PA with +LR (21.2) and −LR (0.15), and of the 43 patients who were SIT indeterminate, the ΔPRA correctly diagnosed 21 patients as PA and 13 patients with EH. Thus, 79.1% patients with inconclusive SIT results received a definitive diagnosis judging by ΔPRA.

PRA was suppressed by SIT in both of PA patients and non-PA patients, while in patients with PA appeared a minor change of PRA during SIT compared to non-PA patients, and it has not been reported in previous literature. This is because PA patients had a low basal renin level, which was further suppressed by sodium loading and volume expansion during SIT [14]. On the other hand, the renin-angiotensin of PA patients suppressed due to long-term feedback regulation of the inappropriately elevated PAC level, which can well explain that whether the renin of PA patients is inhibited by SIT and FST (fludrocortisone suppression test) or stimulated by CCT (captopril challenge test), and the degree of inhibition and stimulation is lower than that of non-PA patients [22–26]. The difference in the degree of PRA suppression between PA and non-PA did provide a certain diagnostic value for the diagnosis of PA in the present study.

The results of our study showed that both of PA patients and non-PA patients had a decrease in PAC and PRA postloading. Compared with non-PA patients, PRA post-SIT was still lower, and PAC post-SIT and ARR post-SIT were still higher in PA patients, which was consistent with other studies [23, 26–28]. There was no difference in the PAC difference before and after SIT (ΔPAC) between the two groups, and the PRA difference before and after SIT (ΔPRA) in the PA group was lower than the non-PA group, which has not been reported before. Among these SIT parameters, PAC post-SIT is a diagnostic indicator of SIT recommended by the guideline. According to ROC analysis, we found that the optimal cutoff value for post-SIT PRA was 0.36 ng/ml/h, which showed a poor diagnostic accuracy compared to the study by Tiu et al. This may be because of the number of subjects included and different diagnostic criteria [26]. The diagnostic performance of ΔPRA was better than post-SIT PRA, and its optimal cutoff value was 0.5 ng/ml/h. Although lack of specificity, ΔPRA provided a strong negative-predictive value in PA diagnosis with NPV of 94.2% and −LR (negative likelihood ratio) of 0.13. The patients with ΔPRA greater than 0.5 ng/ml/h during SIT enable to exclude the diagnosis of PA with −LR of 0.13. Further combined with the optimal cutoff value of post-SIT ARR, ΔPRA was superior to other criteria, with specificity and sensitivity of 97% and 81.6%, which enhanced the diagnostic accuracy of post-SIT ARR, specificity from 90.7% to 97.0%, PPV from 82.1% to 92.6%, and +LR from 9.54 to 21.2 while maintaining the similar NPV (91.6%) and −LR (0.19). Patients with post-SIT ARR more than 34 and ΔPRA less than 0.5 ng/ml/h should be confirmed as PA with +LR (21.2) and −LR (0.15). Overall renin-related indicators including ΔPRA, PRA post-SIT, and ARR post-SIT showed a good diagnostic performance for PA. We cannot compare the diagnostic accuracy of these indicators with PAC post-SIT among all subjects in the present study, while it is worth mentioning that ΔPRA had been of a diagnostic value in patients with an inconclusive SIT result who cannot be definitively diagnosed by PAC post-SIT.

Recommended as one of the four major confirmatory tests by current Endocrine Society guideline, considered by some to be the gold standard suppression test, SIT is widely used because of its safety, feasibility, convenience, and high accuracy [10–13]. As a classic diagnostic indicator of SIT, the optimal cutoff level of PAC post-SIT was a matter of debate and varies from 6 to 11 ng/dl with corresponding sensitivity of 87–90.4% and specificity of 92–95.4% [22–26]. The main controversy was in patients with PAC post-SIT range 5–10 ng/dl, who are considered as with an inconclusive SIT result [10]. For those patients with an inconclusive SIT result, a legacy puzzle of PA diagnosis in our clinical practice, which aroused a general interest of PA research in recent years, a diagnosis of PA is arbitrarily established or rejected based on contextual clinical and biochemical parameters or clarified based on the result of another iterative confirmatory test [28, 29]. The two strategies either lack standardized and objective diagnostic criteria or are cumbersome and difficult to promote. Therefore, numerous centers have tried to find a clear cutoff value, for PAC post-SIT used the FST or a combination of two confirmatory tests as reference standards in their studies [20–22].

We further analysed the clinical data of patients with an inconclusive SIT result, divided them into the PA group and non-PA group, used AVS as reference standards, and found that there was no difference in PAC post-SIT between the two groups (*P*=0.24, *P* < 0.05). It indicated that PAC post-SIT cannot distinguish PA patients from non-PA patients among patients with inconclusive SIT results, which was consistent with Lin C et al.'s study [28]. However, ΔPRA was statistically different between the two groups (*P* < 0.05). After ROC analysis, the AUC of ΔPRA for PA diagnosis was 0.735 (95% CI: 0.602, 0.833, *P* < 0.001), similar to the combination of CCT and SIT in Lin C et al.'s study [28]. At an optimal cutoff value of 0.21 ng/ml/h, according to the highest YI, provide 65.38% sensitivity, 82% specificity, 85% of PPV, and 60.9% of NPV for PA diagnosis. Of the 43 patients who were SIT inconclusive, the ΔPRA ≤ 0.21 ng/ml/h correctly diagnosed 13 patients as EH and 21 patients as PA. Thus, 79% patients with inconclusive SIT results received a definitive diagnosis. Of course, its limited sensitivity revealed us that it is not easy to completely distinguish PA from non-PA among patients with an inconclusive SIT result only based on single parameter of SIT or simple clinical feature (adrenal tumour on CT and hypokalemia). More indicators should be involved just like Velema et al.'s research [29], which established a prediction model for PA diagnosis containing the predictors PRC before and PAC after SIT, and the quantum of potassium supplementation and plasma potassium concentration do well in internal validation. Even maybe a clinical model that included ΔPRA should be established, and it enables a better distinction to be made between those patients with non-PA and patients with PA. ΔPRA had been of a diagnostic value, and the PRA determination after infusion should not be omitted.

### 4.1. Limitations

First, there is no gold standard for PA diagnosis. Even though the FST has been considered the “gold standard,” recent review highlighted the lack of definitive studies that evaluated the diagnostic accuracy of the FST [13]. For subjects considered as PA unlikely or very probable, only those with a surgically resectable UPA can have their diagnoses verified with absolute certainty. We have used the combination of screening ARR and PAC post-SIT or results of AVS as the reference standard in this study. Second, another limitation of this study is that the number of patients with an inconclusive SIT result and relabelled by AVS is relatively small. In order to choose a more objective and accurate reference standard for these patients, we only included patients with perfected AVS. Small number of patients with inconclusive SIT results may affect the results of the study to a certain extent. Third, the diagnostic strategies were currently controversial among patients with inconclusive SIT results, and AVS was conducted for the identification of confirmed PA and unconfirmed PA in this study, which can more precisely and objectively reflect the aldosterone hypersecretion than contextual clinical or another confirmatory test. Finally, as a retrospective study, the diagnostic value of ΔPRA still needs to be further confirmed by a prospective study with FST as the gold standard.

## 5. Conclusions

In conclusion, PA patients show minor PRA change during SIT; change of PRA during SIT provides an auxiliary diagnostic value for PA, especially in patients with inconclusive SIT results, and the PRA determination after infusion has been of a diagnostic value and should not be omitted.

## Figures and Tables

**Table 1:** Comparison of characteristics and SIT parameters in PA and non-PA groups.

	PA (*n* = 159)	Non-PA (*n* = 368)	*P*
Age (years)	46.8 ± 8.8	42.2 ± 8.2	<0.001
Female	117 (63%)	257 (67%)	0.255
BMI (kg/m^2^)	27.1 ± 3.6	27.2 ± 3.7	0.890
Serum potassium (mmol/L)	3.56 ± 0.39	3.76 ± 0.34	0.050
Hypokalemia	42%	20%	<0.001
PAC pre-SIT (ng/dl)	21.5 (17.1, 26.5)	16.5 (13.8, 20.9)	0.001
PRA pre-SIT (ng/ml/h)	0.35 (0.20, 0.59)	1.92 (1.07, 2.43)	<0.001
ARR pre-SIT (ng/dl)/(ng/ml/h)	61.3 (35.3, 84.8)	9.9 (5.9, 15.5)	<0.001
PAC post-SIT (ng/dl)	13.7 (10.8, 18.6)	5.8 (8.2, 11.0)	<0.001
PRA post-SIT (ng/ml/h)	0.20 (0.08, 0.28)	0.66 (0.35, 1.20)	<0.001
ARR post-SIT (ng/dl)/(ng/ml/h)	68.6 (46.3, 94.2)	12.9 (6.7, 20.8)	<0.001
ΔPAC (ng/dl)	6.9 (3.6, 10.8)	8.50 (5.5, 11.7)	0.127
ΔPRA (ng/ml/h)	0.16 (0.05, 0.34)	1.07 (0.52, 1.81)	<0.001
PAC post-SIT/ΔPRA	57.9 (31.4, 121.6)	8.2 (4.2, 16.0)	<0.001

**Table 2:** Diagnostic values for combined criteria using various combinations of SIT parameters.

Criteria	A	B	C	D	E
Post-SIT PRA < 0.36 (ng/ml/h)	***√***				
ΔPRA ≤ 0.5 (ng/ml/h)		***√***			***√***
Post-SIT ARR > 34 (ng/dl)/(ng/ml/h)			***√***		***√***
Post-SIT PAC/ΔPRA > 21 (ng/dl)/(ng/ml/h)				***√***	
Predictive measures: Est. % (95% CI)					
Sensitivity	85.4%	90.3%	89.2%	90.6%	81.6%
	(79.5–90.2)	(85.1–94.1)	(83.8–93.3)	(85.3–94.6)	(75.3–86.9)
Specificity	74.8%	75.6%	90.7%	84.3%	97.0%
	(70.2–79.1)	(71.0–79.8)	(87.3–93.4)	(80.3–87.8)	(94.6–98.4)
PPV	62%	64.0%	82.1%	72.1%	92.6%
	(55.7–67.9)	(57.8–69.8)	(76.1–87.1)	(65.6–78.0)	(88.5–94.1)
NPV	91.4%	94.2%	94.6%	95.3%	91.6%
	(87.8–94.3)	(90.9–96.5)	(91.8–96.7)	(92.4–97.3)	(88.5–94.1)
+L	3.90	3.70	9.54	5.79	26.19
−L	0.20	0.13	0.12	0.11	0.15
Youden	0.602	0.659	0.798	0.749	0.785
AUC	0.855	0.897	0.973	0.893	0.951
	(0.823–0.882)	(0.869–0.920)	(0.960–0.982)	(0.864–0.917)	(0.930–0.967)

Post-SIT ARR, aldosterone-to-renin ratio after the saline infusion test; Post-SIT PRA, plasma renin activity after the saline infusion test; PPV, positive-predictive value; NPV, negative-predictive value; AUC, area under the curve; +L, positive likelihood ratio; −L, negative likelihood ratio. ΔPRA = PRA pre-SIT−PRA post-SIT; PAC post-SIT/ΔPRA = PAC post-SIT/(PRA pre-SIT−PRA post-SIT).

**Table 3:** Characteristics of 43 patients with inconclusive SIT results and completed AVS.

		Confirmed PA	Unconfirmed PA	*P* value
		(*n* = 28)	(*n* = 15)	
Age (years)		47 ± 11.2	45 ± 5.3	0.451
Female		8 (28.7%)	5 (33.3%)	0.746
BMI (kg/m^2^)		27.4 ± 3.9	27.6 ± 3.38	0.844
Systolic (mmHg)		142.8 ± 16.9	134.3 ± 17.5	0.264
Diastolic (mmHg)		94.7 ± 11.0	92.5 ± 6.1	0.636
Hypokalemia (*n*, %)		8 (28.6%)	2 (13.3%)	0.260
PAC^∗^	Pre-SIT	17.1 (13.7–20.1)	15.9 (13.3–19.1)	0.543
(ng/dl)	Post-SIT	7.4 (6.3–8.3)	7.6 (6.8–8.3)	0.118
PRA^∗^	Pre-SIT	0.34 (0.25–0.46)	0.45 (0.34–0.61)	0.054
(ng/ml/h)	Post-SIT	0.17 (0.1–0.25)	0.15 (0.1–0.33)	0.785
ARR^∗^	Pre-SIT	40.6 (28.1–60.2)	38.0 (25.0–44.8)	0.570
	Post-SIT	38.1 (29.7–48.9)	44.8 (22.6–58.2)	0.640
ΔPRA^∗^ (ng/ml/h)		0.16 (0.04–0.28)	0.29 (0.22–0.42)	0.030

Reported values are the number of patients with available data and then number (percentages) or median (first quartile and third quartile) or mean ± standard deviation. Post-SIT PRA, plasma renin activity after the saline infusion test; ΔPRA, decrease values of plasma renin activity after the saline infusion test; Post-SIT ARR, aldosterone-to-renin ratio after the saline infusion test. ^∗^Values are not normally distributed which are given as median (first quartile, third quartile).

## Data Availability

The data used to support the findings of this study are available from the corresponding author upon request.

## Supplementary Materials

Supplementary Materials Supplementary Figure 1: comparison of ROC curves of PRA post-SIT, ΔPRA, and ARR post-SIT with PAC post-SIT for primary aldosteronism (PA) diagnosis. ROC curve of PAC after the saline infusion test (A), ROC curve of PRA after the saline infusion test (B), ROC curve of reduction in PRA during the saline infusion test (C), and ROC curve of aldosterone-renin ratio after the saline infusion test (D) to diagnose PA. Supplementary Table 1: baseline characteristic of included patients.
