# Supplementary material for: Minor Change of Plasma Renin Activity during the Saline Infusion Test Provide an Auxiliary Diagnostic Value for Primary Aldosteronism
Source: Int J Endocrinol. 2021 Feb 17;2021:5757305. doi: 10.1155/2021/5757305 (PMC7904345; doi:10.1155/2021/5757305)
Supplement: Supplementary Materials — Supplementary Figure 1: comparison of ROC curves of PRA post-SIT, ΔPRA, and ARR post-SIT with PAC post-SIT for primary aldosteronism (PA) diagnosis. ROC curve of PAC after the saline infusion test (A), ROC curve of PRA after the saline infusion test (B), ROC curve of reduction in PRA during the saline infusion test (C), and ROC curve of aldosterone-renin ratio after the saline infusion test (D) to diagnose PA. Supplementary Table 1: baseline characteristic of included patients. [file 5757305.f1.zip › 5757305.f1/Supplementary Figure 1.pdf]

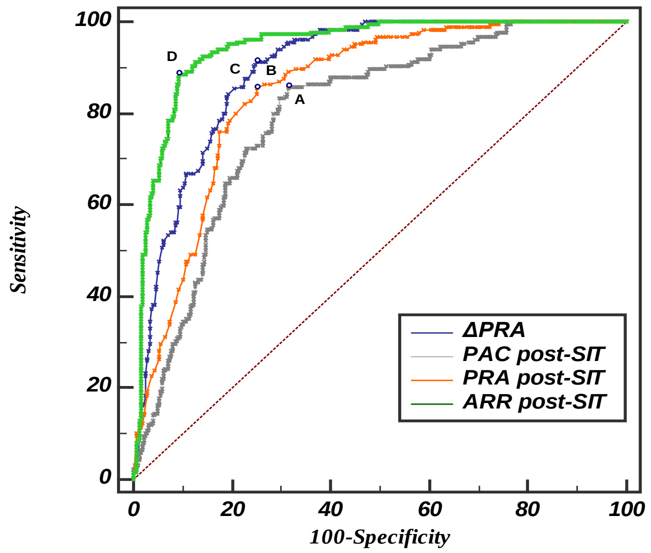

Supplementary Figure 1. Comparison of ROC curves of PRA post-SIT, ΔPRA, ARR post-SIT with PAC post-SIT for primary aldosteronism (PA) diagnosis. ROC curve of PAC after saline infusion test(A); ROC curve of PRA after saline infusion test(B); ROC curve of reduction in PRA during saline infusion test(C); ROC curve of aldosterone–renin ratio after saline infusion test(D) to diagnose PA.
