# Supplementary material for: Minor Change of Plasma Renin Activity during the Saline Infusion Test Provide an Auxiliary Diagnostic Value for Primary Aldosteronism
Source: Int J Endocrinol. 2021 Feb 17;2021:5757305. doi: 10.1155/2021/5757305 (PMC7904345; doi:10.1155/2021/5757305)
Supplement: Supplementary Materials — Supplementary Figure 1: comparison of ROC curves of PRA post-SIT, ΔPRA, and ARR post-SIT with PAC post-SIT for primary aldosteronism (PA) diagnosis. ROC curve of PAC after the saline infusion test (A), ROC curve of PRA after the saline infusion test (B), ROC curve of reduction in PRA during the saline infusion test (C), and ROC curve of aldosterone-renin ratio after the saline infusion test (D) to diagnose PA. Supplementary Table 1: baseline characteristic of included patients. [file 5757305.f1.zip › 5757305.f1/Supplementary Table 1.docx]

Supplementary Table 1. Baseline Characteristic of Included Patients

| Variable | |  | Subjects |
| --- | --- | --- | --- |
| Age, (years) | |  | 43.8±9 |
| Female | |  | 379(66%) |
| BMI, (kg/m2) | |  | 27.1±3.6 |
|  | office blood pressure measurement | | |
| Systolic, (mmHg) | |  | 143±19.5 |
| Diastolic, (mmHg) | |  | 97±13.8 |
|  | Laboratory measurements | | |
| PAC*, (ng/dl) | |  | 17.8 (14.5 – 23.7) |
| PRA*, (ng/ml/h) | |  | 1.19 (0.42 – 2.49) |
| ARR*, (ng/dl)/(ng/ml/h) | |  | 15.0 (7.6 – 44.0) |
| Serum potassium, (mmol/l) | |  | 3.69±0.38 |
| Hypokalemia (n, %) | |  | 155 (27%) |
| ARR≥20 (n, %) | |  | 236 (41%) |
| PA (n, %) | |  | 185 (32%) |

Reported values are the number of patients with available data and then number (percentages) or median [first quartile, third quartile] or mean ± standard deviation. ARR, aldosterone-to-renin ratio; BMI indicates body mass index; PA, primary aldosteronism. PAC, plasma aldosterone concentration; PRA, plasma renin activity; Serum K+, concentration of serum potassium. (*) Values that are not normally distributed are given as median [first quartile, third quartile].
